# Supplementary material for: A comprehensive structural, biochemical and biological profiling of the human NUDIX hydrolase family
Source: Nat Commun. 2017 Nov 16;8:1541. doi: 10.1038/s41467-017-01642-w (PMC5688067; doi:10.1038/s41467-017-01642-w)
Supplement: Supplementary file 1 — Supplementary Information [file 41467_2017_1642_MOESM1_ESM.pdf]

Supplementary Information

Supplementary Figures:

a

	% Pairwise Identity	Patristic distance
NUDT3, NUDT4, NUDT10, NUDT11	91.9	0.11
NUDT7, NUDT8, NUDT16, NUDT19	19.4	2
NUDT7, NUDT8	36	1.27
NUDT12, NUDT13	41.7	0.87
NUDT5, NUDT14	18.1	1.96
MTH1, NUDT15	24.6	2.45
NUDT2, NUDT21	18.2	2.01
Average	35.7	2.88

b

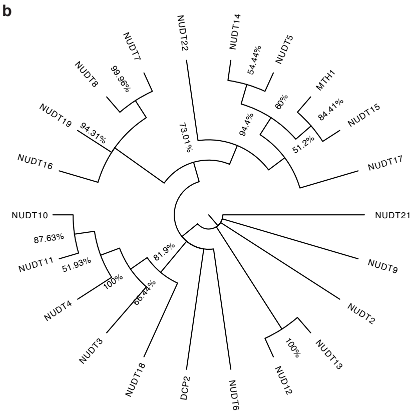

c

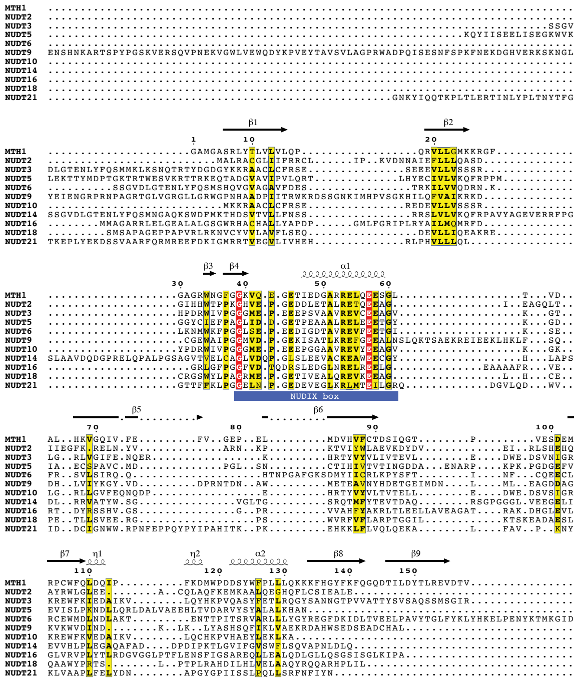

Supplementary Figure 1

Supplementary Figure 1: A. % Pairwise distances and Patristic distances for each branch of the consensus phylogenetic tree of NUDIX full length sequences of the human NUDIX enzymes. B. Consensus phylogenetic tree of NUDIX fold sequences of the human NUDIX proteins with % pairwise distances of each branch provided. B**.** Sequence alignment of human NUDIX hydrolases with known structure, homology is highlighted in yellow, strict sequence conservation in red, and the NUDIX box is indicated in blue.

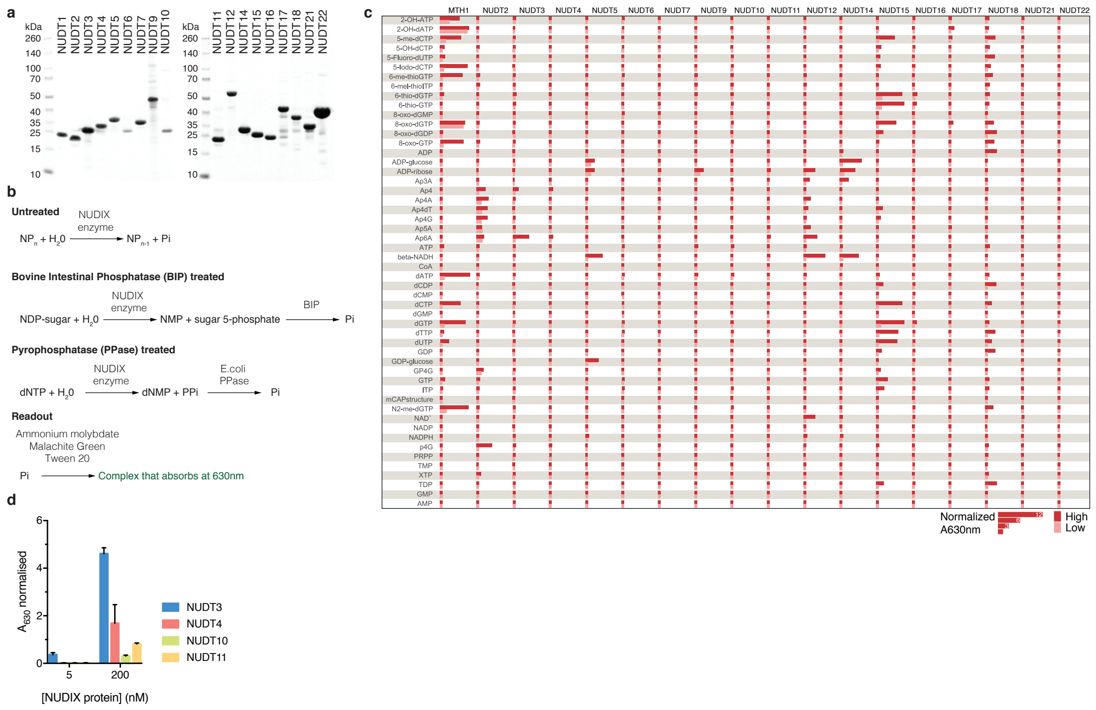

Supplementary Figure 2: NUDIX enzymes purification, substrate screen principle and DIPPs activity. A: 4 μg of purified NUDIX protein was analyzed on SDS-PAGE and stained using Coomassie Blue. B: Principles behind the substrate screen, which uses the Malachite Green reaction as read-out. C: Activity of NUDT3, NUDT4, NUDT10 and NUDT11 with 5-PP-InsP5 as substrate. D: Activity of 18 human NUDIX hydrolases towards 52 substrates. Activity is represented by a column bar graph in which the absorbance at 630nm normalized to untreated controls (this is, without BIP or PPase) is shown. Light-red bars indicate activity in conditions of low enzyme concentrations (5 nM), while dark-red bars indicate activity at high enzyme concentration (200 nM).

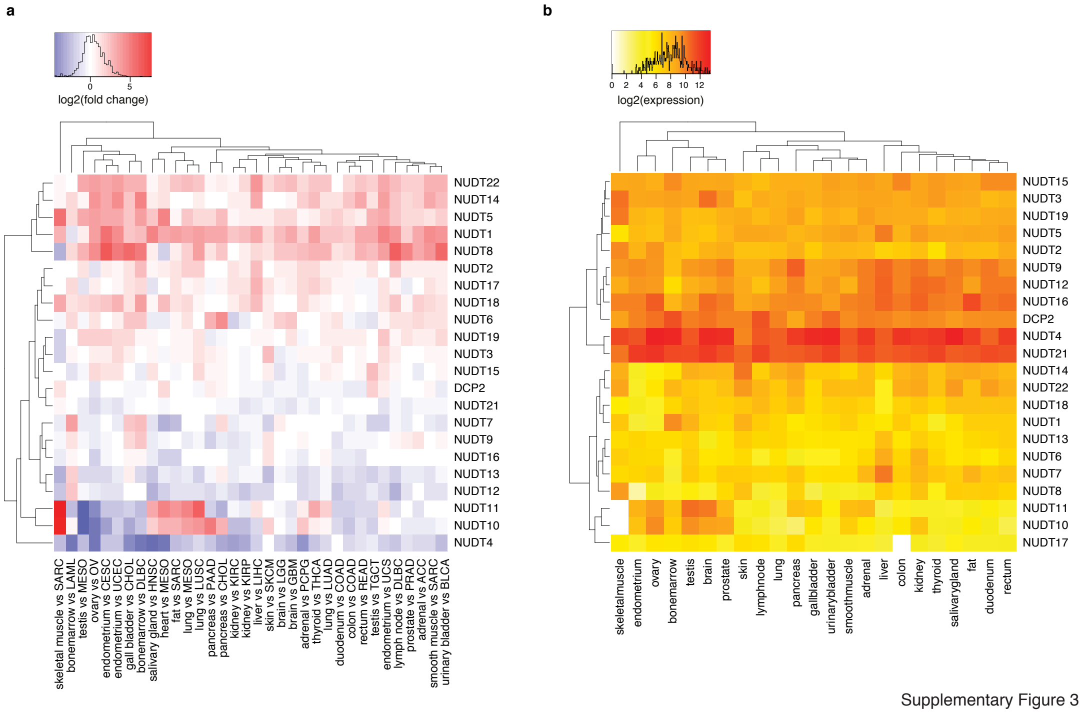

Supplementary Figure 3: A: Clustering of the NUDIX hydrolases by mRNA expression by comparing normal tissues with their cancer counterparts. B: Clustering of the NUDIX hydrolases by their mRNA expression in normal tissues.

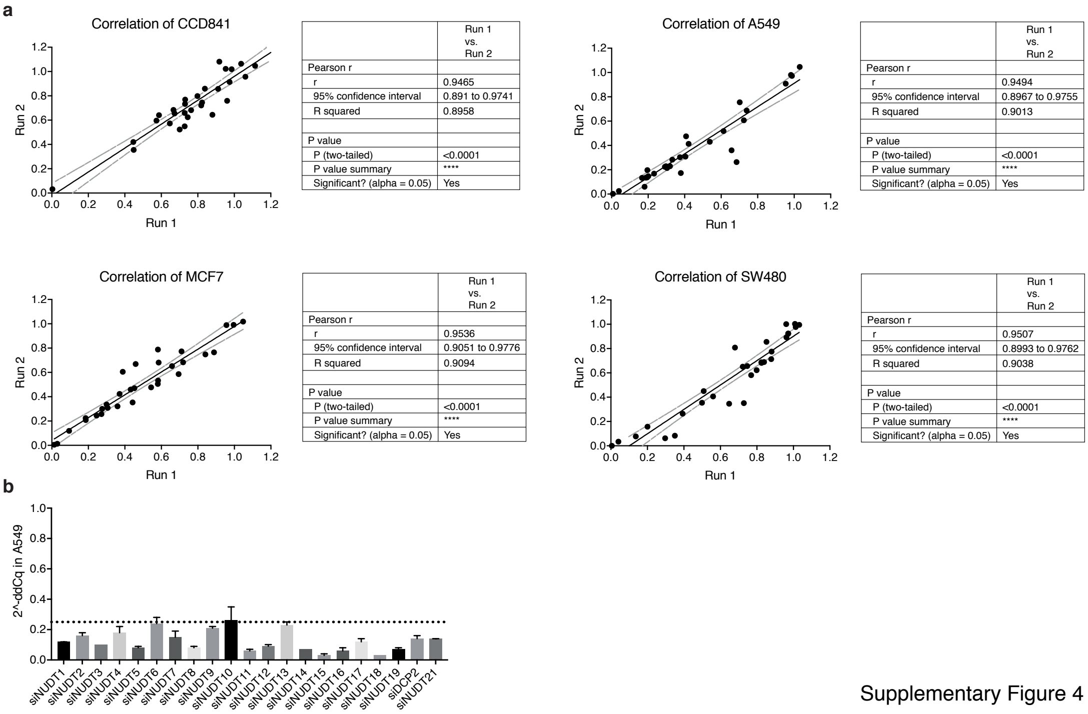

CCD841:

	Run 1 vs. Run 2
Pearson r	
r	0.9465
95% confidence interval	0.891 to 0.9741
R squared	0.8958
P value	
P (two-tailed)	<0.0001
P value summary	****
Significant? (alpha = 0.05)	Yes

A549:

	Run 1 vs. Run 2
Pearson r	
r	0.9494
95% confidence interval	0.8967 to 0.9755
R squared	0.9013
P value	
P (two-tailed)	<0.0001
P value summary	****
Significant? (alpha = 0.05)	Yes

MCF7:

	Run 1 vs. Run 2
Pearson r	
r	0.9536
95% confidence interval	0.9051 to 0.9776
R squared	0.9094
P value	
P (two-tailed)	<0.0001
P value summary	****
Significant? (alpha = 0.05)	Yes

SW480:

	Run 1 vs. Run 2
Pearson r	
r	0.9507
95% confidence interval	0.8993 to 0.9762
R squared	0.9038
P value	
P (two-tailed)	<0.0001
P value summary	****
Significant? (alpha = 0.05)	Yes

Supplementary Figure 4: A: Statistical correlation between two independent siRNA knockdown experiments per cell line. B: mRNA expression of the different human NUDIX genes upon siRNA-mediated depletion, measured by qRT-PCR in intra-assay triplicates.

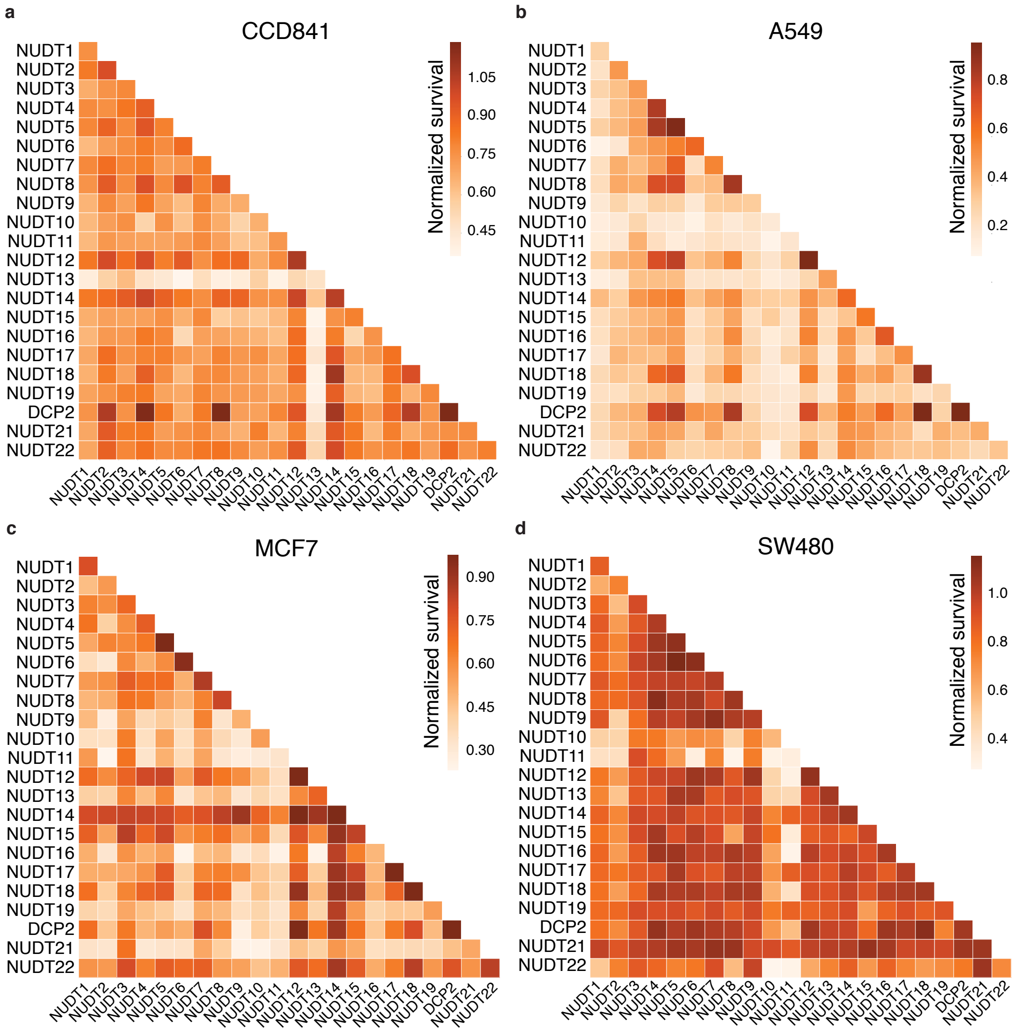

Supplementary Figure 5

Supplementary Figure 5: Quantitative scores for cell viability of double NUDIX knockdowns corresponding to (a) CCD841, (b) A549, (c) MCF7 and (d) SW480 respectively and normalized to the corresponding single knockdown.

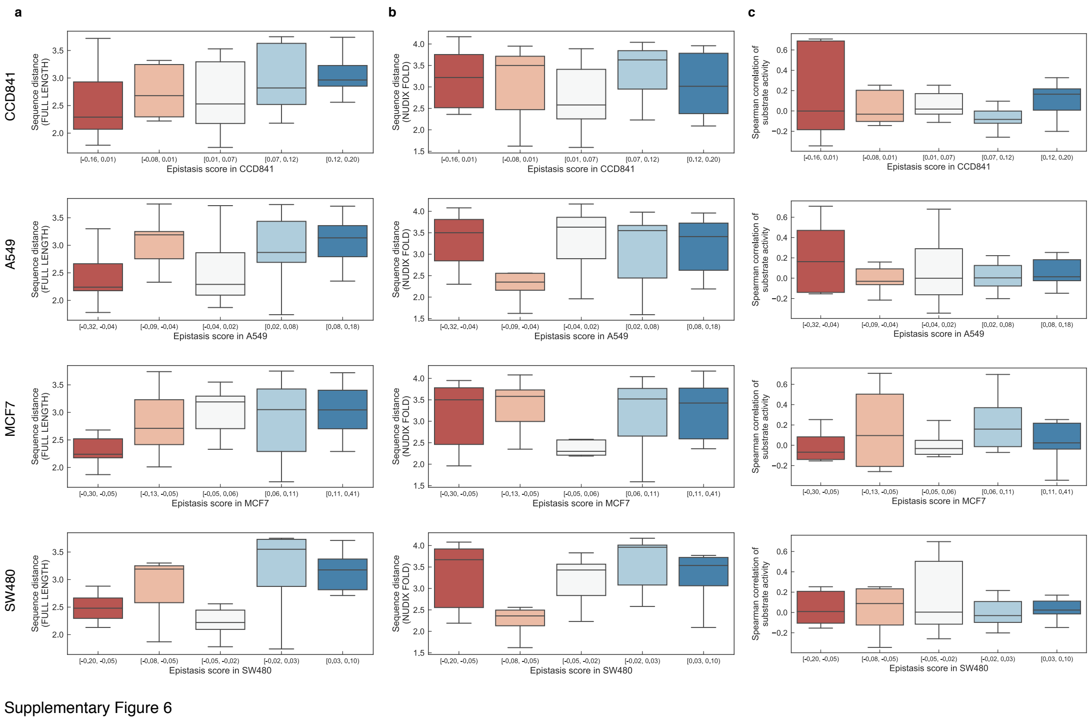

Supplementary Figure 6: A: Box plot comparing Sequence Patristic distance of full length NUDIX sequence with epistasis scores. B: Box plot comparing Sequence Patristic distance of NUDIX fold sequence with epistasis scores. C: Box plot comparing Spearman rank correlation of substrate activity similarity with epistasis scores.

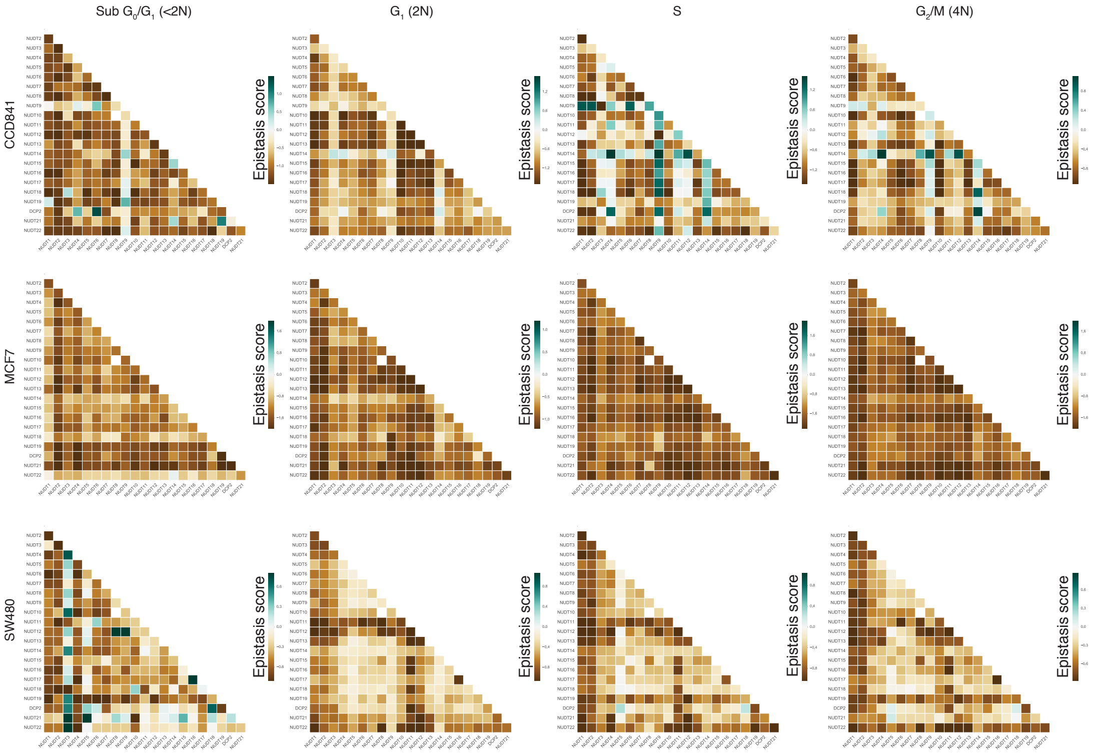

Supplementary Figure 7

Supplementary Figure 7: Cell cycle based epistasis scores between NUDIX genes in CCD841, MCF7 and SW480 cells. The interaction maps visualize interactions determined based on the fraction of pairwise siRNA depleted cells in the cell cycle distribution. The panels show one interaction map per cell cycle phase. For each cell cycle phase, an interaction was assigned to a pair of genes if the cell fraction of the double knockdown was significantly different from the cell fraction of the double knockdown that would be expected if the genes were not interacting. The expected cell fraction was determined with a multiplicative null function. The interaction maps include negative (or decrease in cell fraction) interactions in brown, as well as positive (or increase in cell fraction) interactions in green. Positive interactions suggest that certain NUDIX product operate in concert or in series within the same pathway.

CCD841 - Cell Cycle

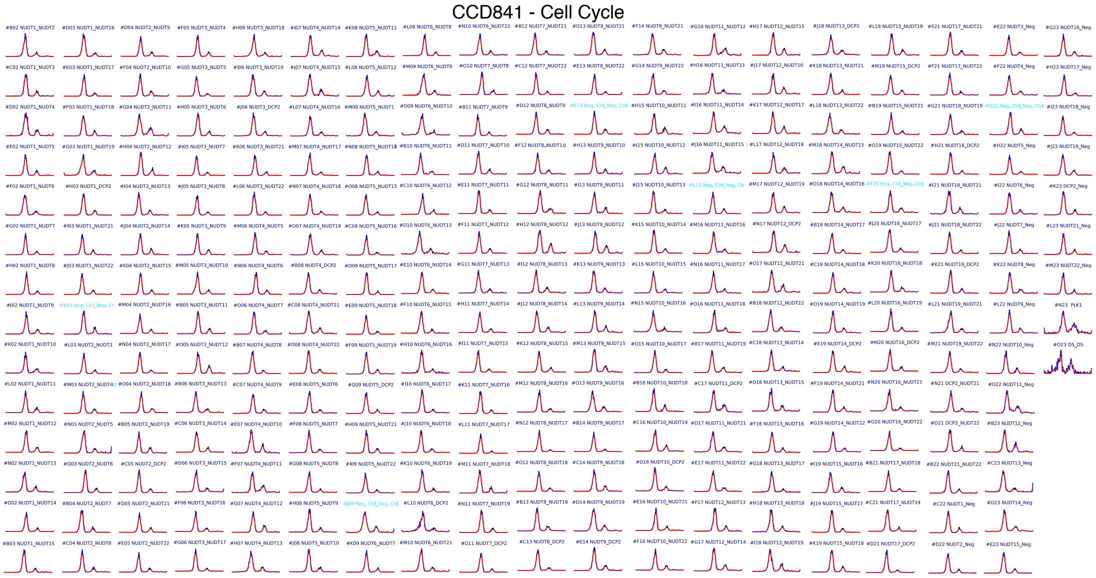

Supplementary Figure 8

A549 - Cell Cycle

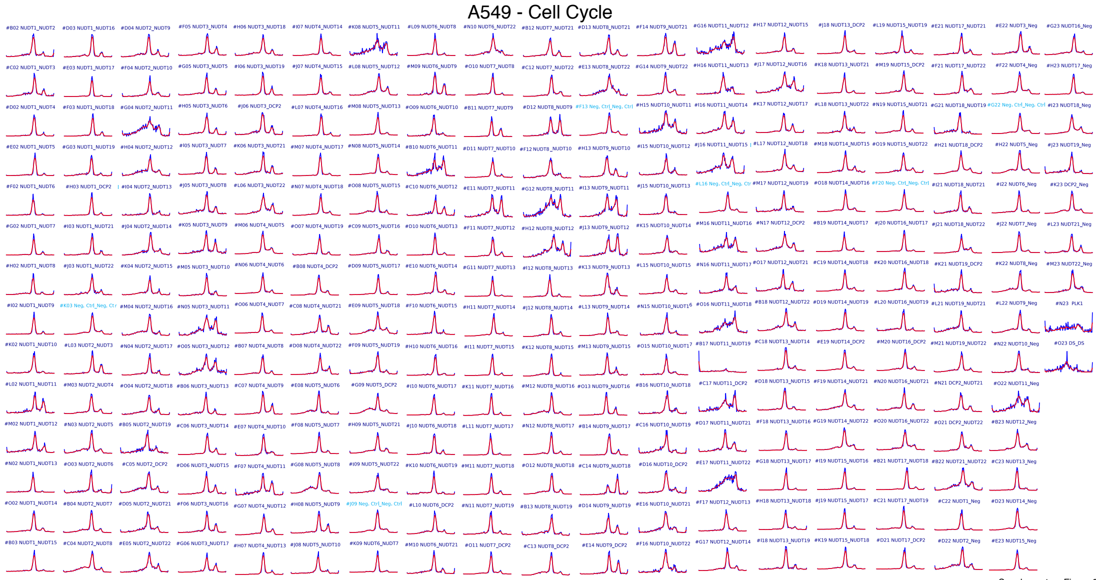

Supplementary Figure 9

MCF7 - Cell Cycle

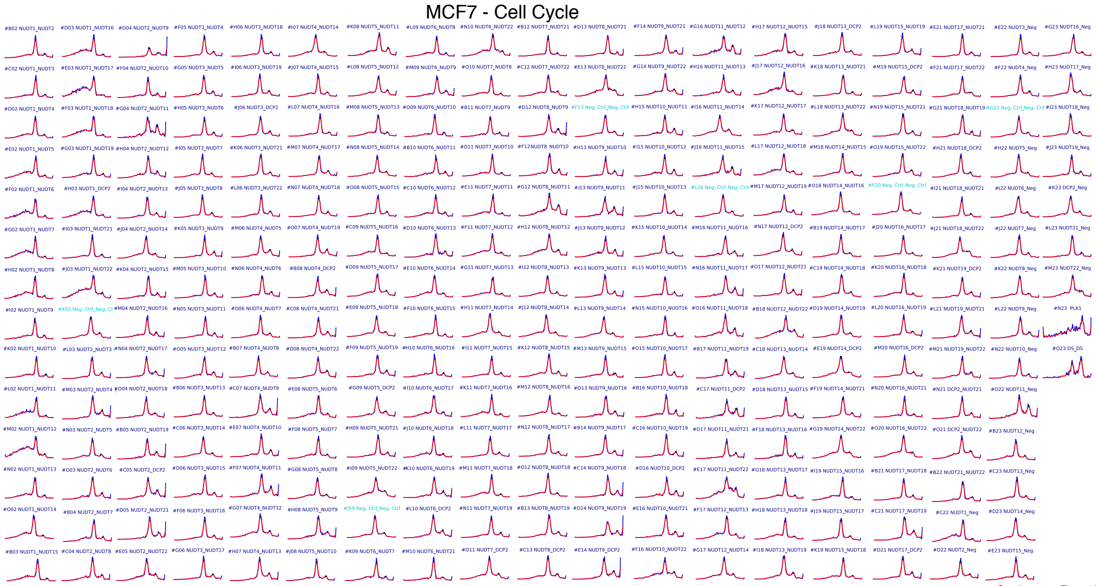

Supplementary Figure 10

SW480 - Cell Cycle

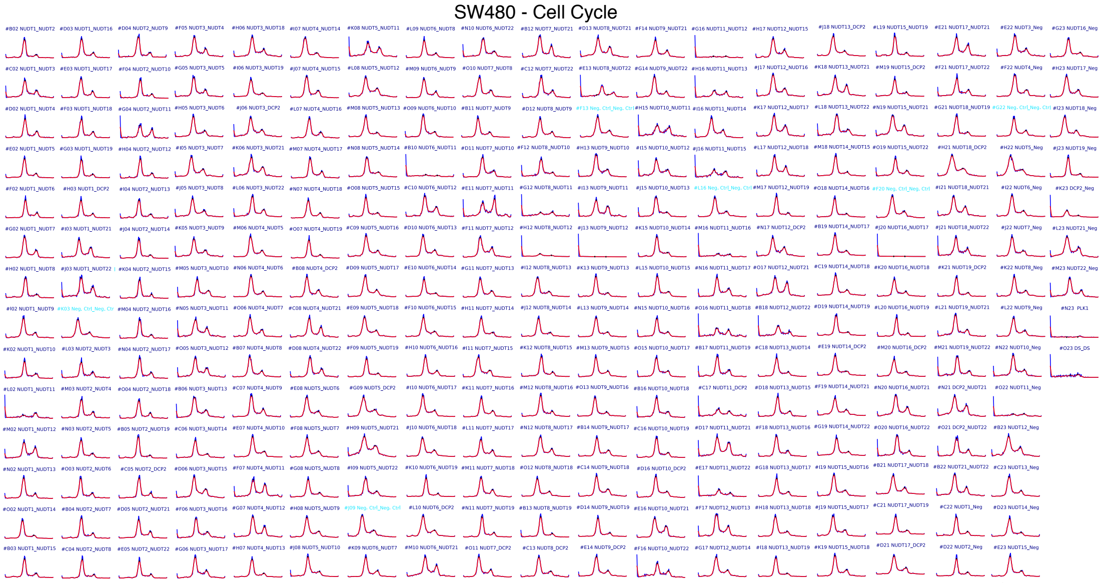

Supplementary Figure 11

Supplementary Figures 8 to 11: Cell cycle histograms of CCD841, A549, MCF7 and SW480 upon double NUDIX depletion. The graphical representation of the cell cycle histograms was obtained using PopulationProfiler.

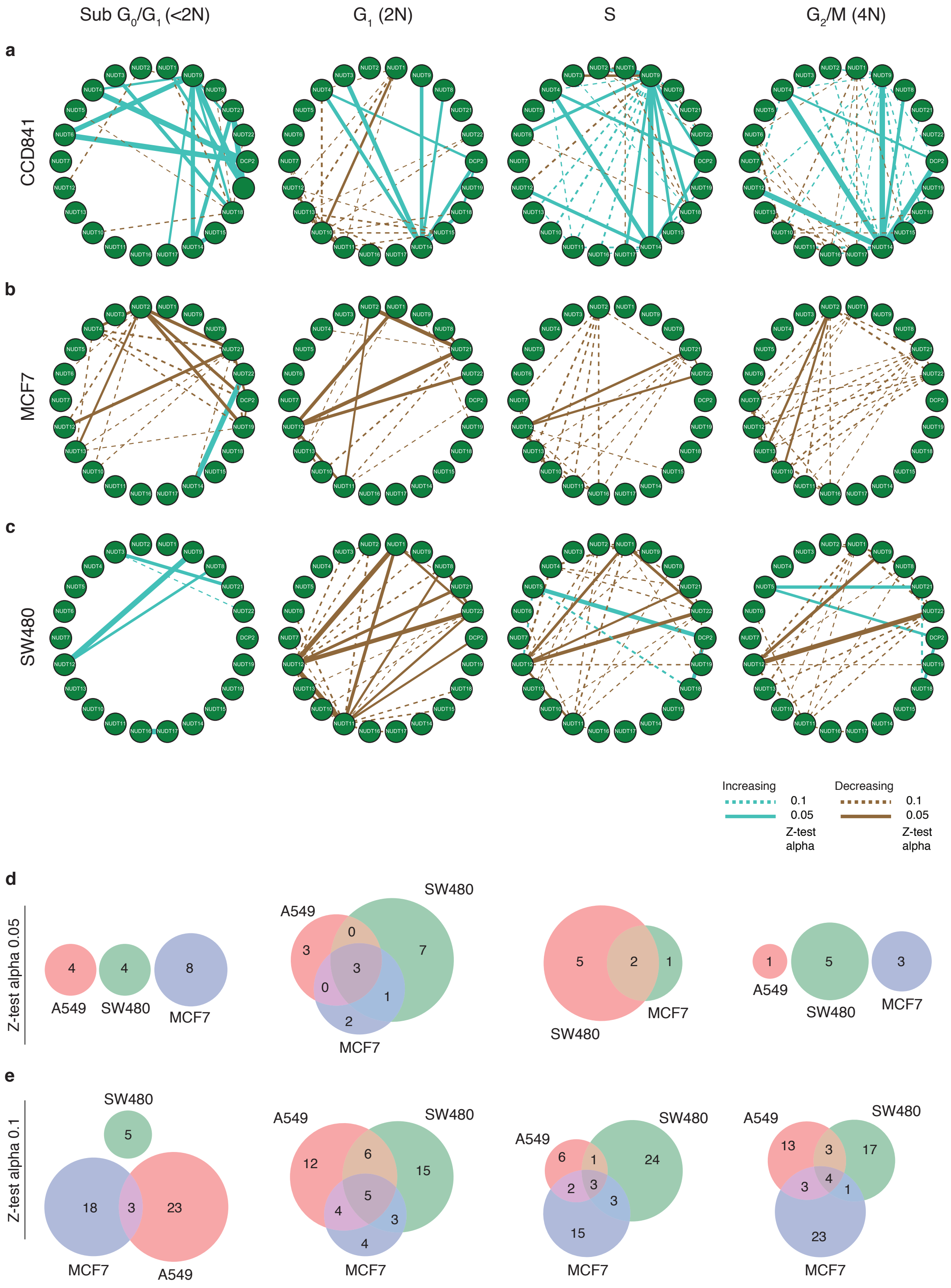

Supplementary Figure 12

Supplementary Figure 12: A to C: Selected cell cycle based interactions between NUDIX genes in CCD841, MCF7 and SW480 cells are visualized using circular networks. The panel shows one network per cell cycle phase. For each gene pair, the interaction was assessed by using a two-tailed Z test (indicated by a dotted line for alpha = 0.1, or solid line for alpha = 0.05). Shown are interactions whose values are significantly larger (positive

interaction with increase in cell fraction) in green, or significantly smaller (negative interaction with decrease in cell fraction) in brown, than values in the 90 % of interaction probability density. The interactions were selected independently and separately for each cell cycle phase in CCD841, MCF7 and SW480 cells. D and E: The overlap of significant genetic interactions from panels A, B and C is shown using Venn diagrams. The size of each circle in the diagram is proportional to the number of significant genetic interactions in the respective cell line.

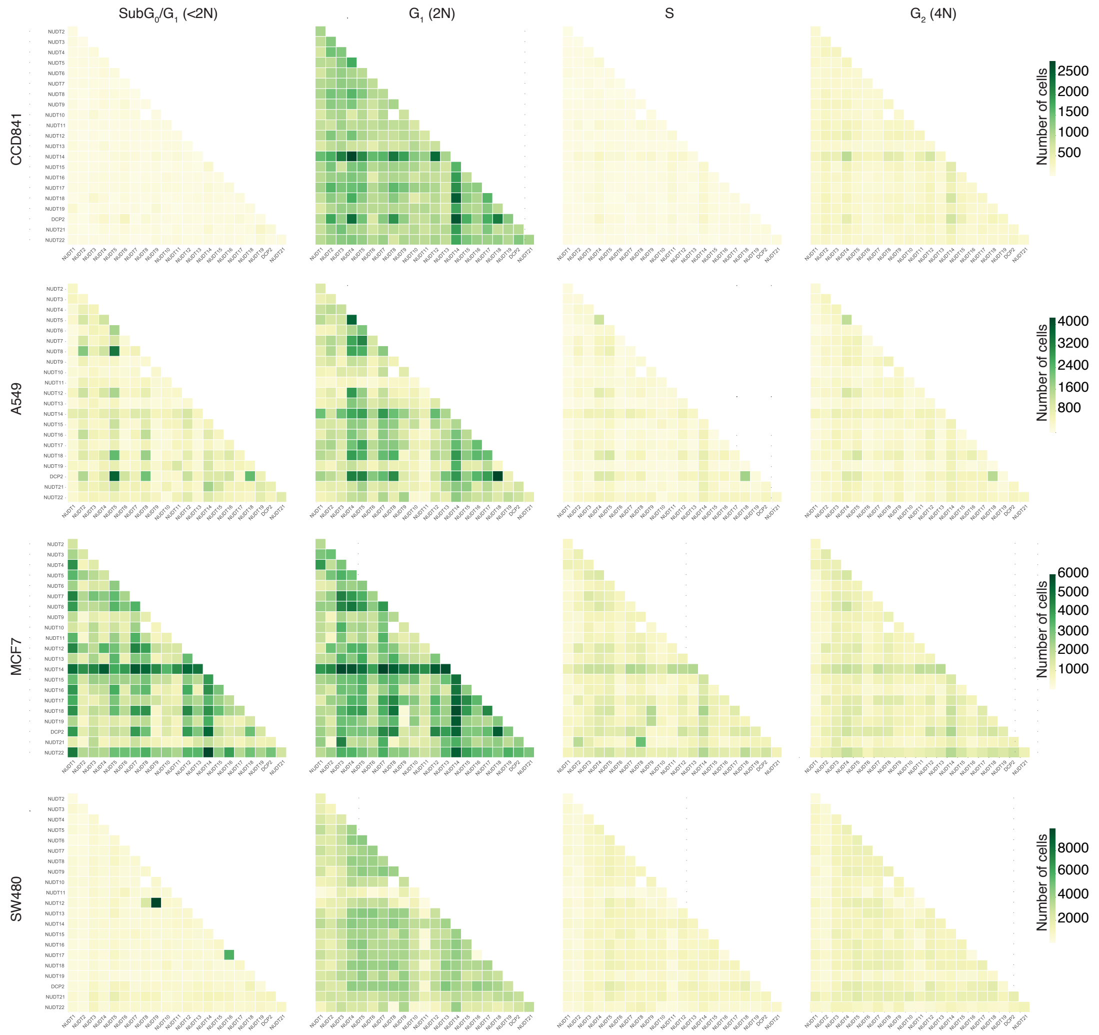

Supplementary Figure 13

Supplementary Figure 13: Cell cycle distributions in double NUDIX knockdown. Heat map representations of the cell numbers in each cell cycle phase (<2N ($SubG_0/G_1$), 2N (G_1), S, 4N (G_2-M)) as determined using PopulationProfiler.

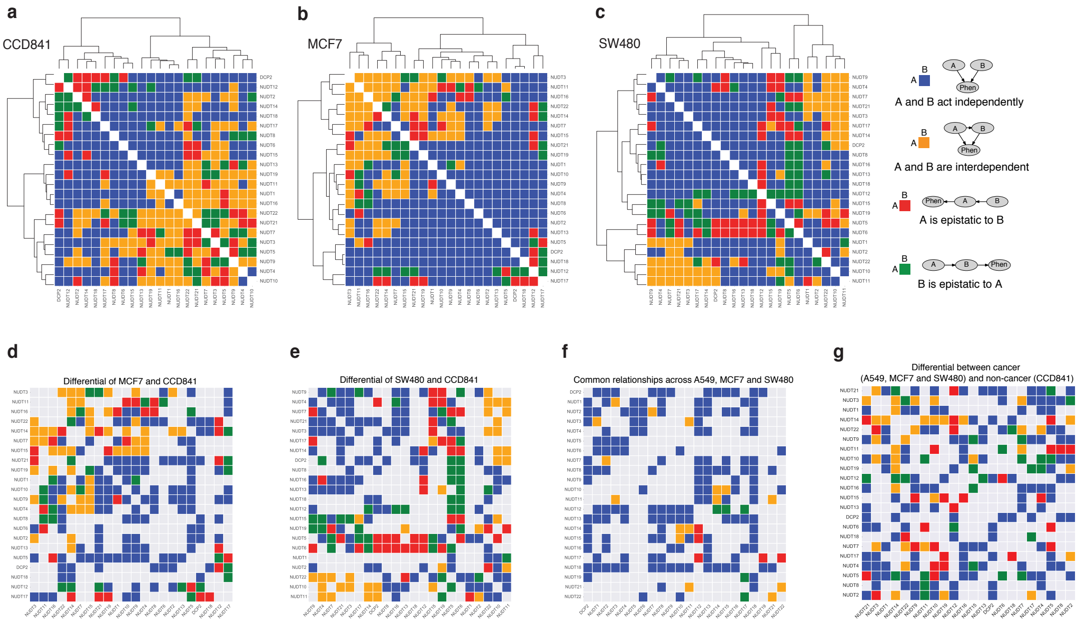

Supplementary Figure 14

Supplementary Figure 14: A, B and C: Many gene-gene relationships corresponding to independent activity in parallel pathways arise repeatedly when analysing knockdown phenotype data from different cancers. In addition to being interested in genetic relationships that persist across cancers we modelled cell viability data from individual cancer cell lines. D and E: Differential gene-gene relationships between MCF7, SW480 and CCD841, respectively. F: Common genetic interactions in all three cancer cell lines. G: Differential genetic interactions between all three cancer cell lines and the non-cancer cells CCD841. The space of cancer-specific gene-gene relationships shows substantial deviation from the space of corresponding relationships estimated from non-cancer cell data

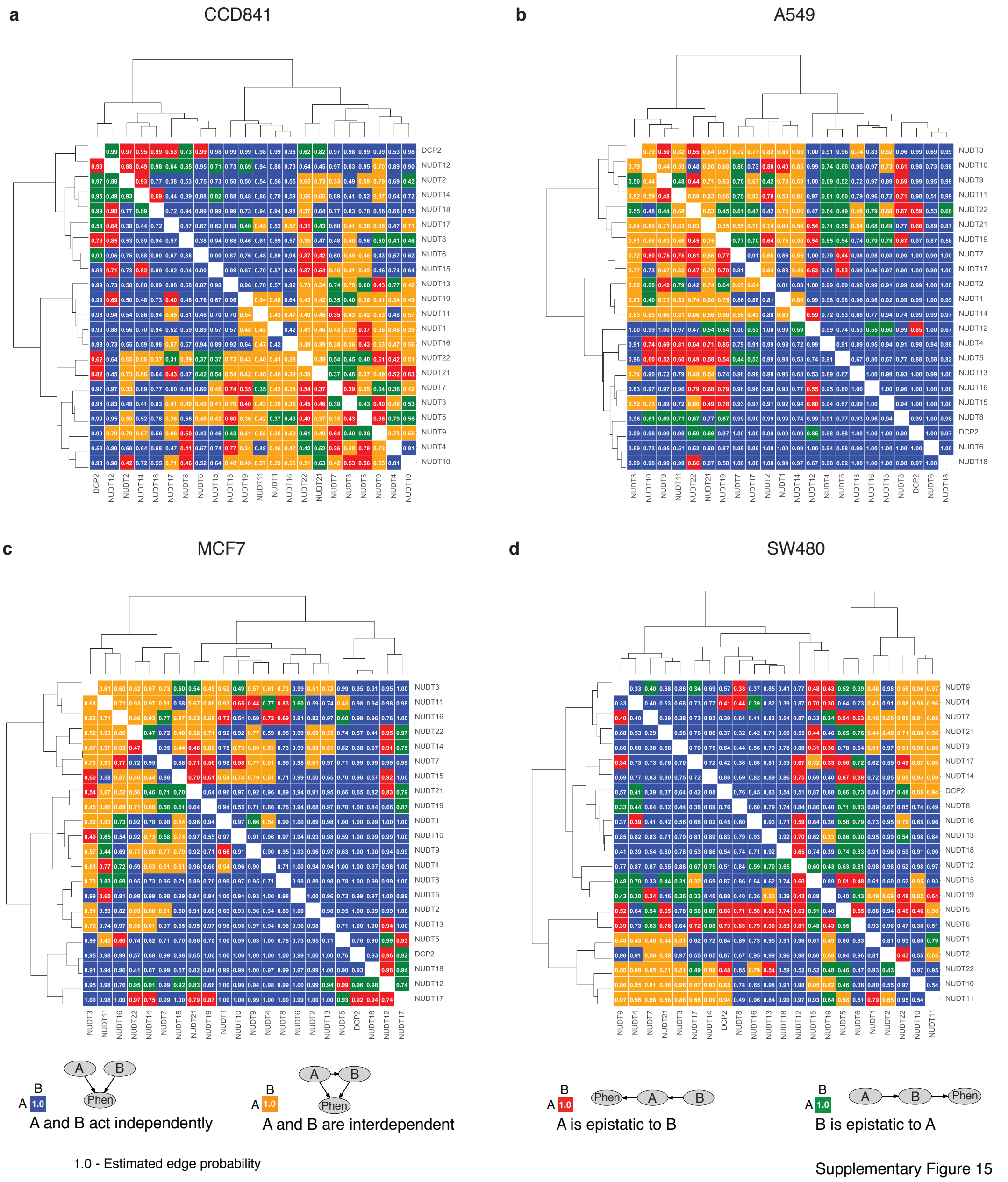

Supplementary Figure 15: Probabilistic scoring of relationships from data on cell viability of single and double NUDIX knockdowns. We fed cell line-specific cell viability data to Réd and estimated probabilistic scores that predicted different types of pairwise NUDIX gene relationships. Prediction of gene-gene relationships involves estimating probabilities for four types of interaction, which are colour-coded in the figure: upstream epistasis, downstream epistasis, parallel pathway and partial interdependence. For a given cell line and a NUDIX gene pair, we show a relationship that was estimated with the highest probability by Réd and was also stable under small data perturbation. Numbers in the heat maps are probabilistic estimates for these most likely relationships. We further performed a hierarchical clustering on the heat maps using the Hamming distance as a measure of discordance between estimated

gene interaction profiles. Rows and columns of the heat maps were reordered according to clustering results.

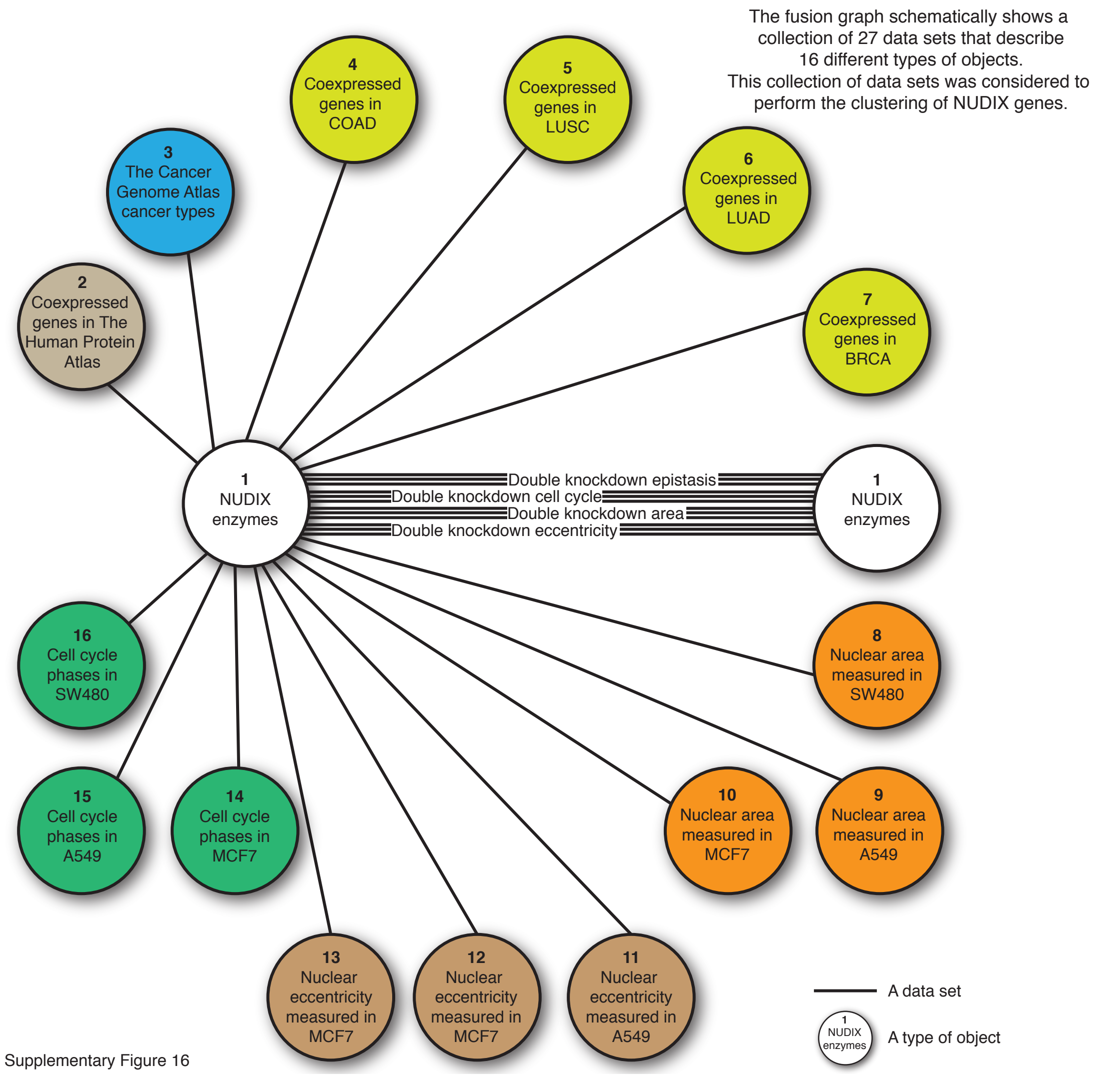

Supplementary Figure 16: Data fusion graph for mining data on NUDIX enzymes. The graph shows the organizational structure of data sets considered for our integrative clustering study. A node in the graph corresponds to a distinct object type. For example, the node named "The Cancer Genome Atlas cancer types" subsumes all cancer types from the TCGA (see Supplementary Data 2 - TCGA Cancer list and abbreviations), and node "Cell cycle phases in SW480" contains five phases of cell cycles, <2N ($SubG_0/G_1$), 2N (G_1), S, 4N (G_2-M). See Supplementary Data 3 for a detailed description of all data sets considered in the analysis. Edges in the graph correspond to data sets. The data fusion algorithm used to cluster NUDIX enzymes treats each data set as a data matrix, whose rows correspond to objects contained in one node of the respective edge and columns representing objects from the other node of the edge. For example, the edge from "NUDIX genes" to "Co-expressed genes in COAD" is technically a data matrix whose element in the *i*th row and *j*th column indicates the strength of co-expression between *i*th NUDIX gene and the *j*th gene in the TCGA COAD cancer type. To model data related to double

knockdowns we virtually doubled the “NUDIX genes” node while keeping its interacting data sets fixed. In total, our integrative data analysis considered 16 different object types and 27 different data sets.

Results summary tables

a

	Structure similarity with
NUDT1	NUDT15
NUDT15	NUDT1
NUDT2	NUDT21
NUDT21	NUDT2
NUDT3	NUDT4, NUDT10, NUDT11, NUDT6
NUDT4	NUDT3, NUDT10, NUDT11, NUDT6
NUDT6	NUDT3, NUDT4, NUDT10, NUDT11
NUDT10	NUDT3, NUDT4, NUDT11, NUDT6
NUDT11	NUDT3, NUDT4, NUDT10, NUDT6
NUDT5	NUDT14
NUDT14	NUDT5
NUDT7	NUDT8
NUDT8	NUDT7
NUDT12	NUDT13
NUDT13	NUDT12
NUDT9	-
NUDT16	-
NUDT17	-
NUDT18	-
NUDT19	-
DCP2	-
NUDT22	-

b

	**Substrates** (newly identified susbtrates*)		
	Low enzyme concentration	High enzyme concentration	Substrate similarity with
NUDT1	2-OH-dATP, 8-oxo-dGTP, 2-OH-ATP, N2-me-dGTP*, 5-me-dCTP	dATP, 5-Iodo-dCTP, dGTP, 8-oxo-GTP, 6-me-thioGTP, dCTP, 2-OH-ATP, dUTP, 5-Fluoro-dUTP, GTP	NUDT15, NUDT18
NUDT2	Ap6A, Ap5A, Ap4dT*, GP4G, Ap4G*	Ap4*, p4G*	NUDT3
NUDT3	-	Ap4, Ap6A	NUDT2
NUDT5	ADP-glucose, ADP-ribose	beta-NADH, GDP-glucose	NUDT9, NUDT12, NUDT14
NUDT9	ADP-ribose	-	NUDT5, NUDT12, NUDT14
NUDT12	-	beta-NADH, Ap6A, ADP-ribose, NAD^+, Ap5A, Ap4A, Ap3A, Ap4dT	NUDT5, NUDT9, NUDT14
NUDT14	-	ADP-glucose, beta-NADH*, ADP-ribose, Ap3A*	NUDT5, NUDT9, NUDT12
NUDT15	6-thio-dGTP, 6-thio-GTP, dGTP	5-me-dCTP, dCTP, 8-oxo-dGTP, dUTP, GTP, ITP, TDP, 8-oxo-dGDP, GDP, dCDP, 5-OH-dCTP	NUDT1, NUDT18
NUDT17	-	2-OH-dATP	NUDT1
NUDT18	-	8-oxo-dGDP, ADP, dCDP, dTTP, 5-me-dCTP, GDP, 5-Fluoro-dUTP, TDP, 8-oxo-GTP, 8-oxo-dGTP, N2-me-dGTP, 6-methyl-thio GTP, dCTP, dUTP, 5-Iodo-dCTP, ATP, 6-methyl-thio ITP, 5-OH-dCTP, 6-thio-dGTP, GTP, 6-thio-GTP, ITP, dGTP	NUDT1, NUDT15

c

	**Protein expression in normal tissues**		
	High	Medium	Low
NUDT1	Colon, Kidney, Stomach	Pancreas, Prostate, Testis, Urinary bladder	Breast, Glial cells, Melanocytes
NUDT5		Liver	Kidney, Testis
NUDT7	Breast, Pancreas, Kidney, Urinary bladder	Colon, Liver, Prostate, Keratinocytes, Stomach	Melanocytes, Testis
NUDT8	Colon, Kidney	Breast, Endometrium, Liver, Melanocytes, Pancreas, Prostate, Keratinocytes, Stomach, Testis, Urinary bladder	Glial cells
NUDT9	Colon, Pancreas, Kidney	Breast, Endometrium, Glial cells, Liver, Melanocytes, Prostate, Keratinocytes, Stomach, Testis, Urinary bladder	
NUDT12	Colon, Melanocytes, Pancreas, Kidney, Stomach, Testis, Urinary bladder	Breast, Endometrium, Glial cells, Prostate	Liver
NUDT13		Colon, Stomach	Breast, Keratinocytes, Testis
NUDT14	Melanocytes, Pancreas, Kidney, Keratinocytes, Urinary bladder	Breast, Colon, Endometrium, Prostate, Stomach, Testis	Liver
NUDT15	Breast, Colon, Liver, Prostate, Kidney, Stomach, Testis, Urinary bladder	Endometrium, Glial cells, Melanocytes, Pancreas	Keratinocytes
NUDT16		Breast, Pancreas, Kidney, Keratinocytes, Stomach	Colon, Liver, Melanocytes, Testis, Urinary bladder
NUDT17	Colon, Testis, Urinary bladder	Breast, Endometrium, Pancreas, Prostate, Kidney, Stomach	Melanocytes, Keratinocytes
NUDT18		Colon, Keratinocytes, Urinary bladder	Breast, Glial cells, Melanocytes, Prostate, Kidney, Stomach, Testis
DCP2		Breast, Colon, Endometrium, Gliacl cells, Liver, Pancreas, Prostate, Kidney, Stomach, Testis, Urinary bladder	
NUDT22	Pancreas	Colon, Liver, Kidney, Stomach, Testis	Breast, Melanocytes, Prostate, Keratinocytes, Urinary bladder

d

	**Survival (%) upon knockdown**			
	CCD841	SW480	MCF7	A549
NUDT1	59.7	49.9	54.3	16.6
NUDT2	75.9	39.3	27.6	33.3
NUDT3	68.5	50.8	45.2	37.4
NUDT4	91.5	88.0	43.2	53.8
NUDT5	79.9	68.0	45.9	73.9
NUDT6	54.9	96.2	29.9	32.0
NUDT7	73.4	82.4	58.2	29.2
NUDT8	85.7	87.9	58.0	61.3
NUDT9	65.9	79.8	18.6	19.6
NUDT10	68.2	29.7	27.2	18.0
NUDT11	65.5	13.6	24.5	19.4
NUDT12	100.0	72.8	71.1	68.4
NUDT13	35.6	72.3	44.2	23.2
NUDT14	100.0	82.5	88.8	40.8
NUDT15	64.4	64.8	69.4	65.7
NUDT16	52.4	85.2	36.0	42.2
NUDT17	74.5	74.7	38.8	29.8
NUDT18	72.2	97.2	84.1	72.4
NUDT19	62.5	55.9	30.6	18.8
DCP2	100.0	97.1	58.5	95.3
NUDT21	64.2	76.8	18.5	37.9
NUDT22	76.9	35.1	65.9	30.4

e

Sub-cellular localisation

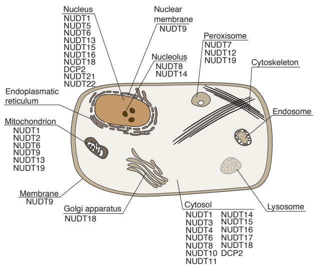

f

	**Réd interactions (directional)**	
	CCD841	A549-MCF7-SW480
NUDT1	NUDT13	NUDT22
NUDT2	NUDT9	
NUDT3		NUDT1
NUDT5		NUDT18
NUDT6	NUDT1	
NUDT7		NUDT13
NUDT8	NUDT21	NUDT17, NUDT22
NUDT9		NUDT2
NUDT10	NUDT9	NUDT11, NUDT21
NUDT11		NUDT21
NUDT12		DCP2, NUDT9
NUDT13		NUDT16
NUDT14	NUDT13	NUDT6, NUDT17
NUDT15	NUDT16	NUDT13
NUDT16	NUDT13	NUDT15
NUDT17		NUDT4, NUDT6, NUDT16
NUDT18		NUDT8, NUDT11, NUDT14
DCP2		NUDT5, NUDT18
NUDT21		NUDT9

g

	**FUSION clustering**
NUDT1	
NUDT2	NUDT3
NUDT3	NUDT2
NUDT4	NUDT5, NUDT6, NUDT7, NUDT8, NUDT9
NUDT5	NUDT4, NUDT6, NUDT7, NUDT8, NUDT9
NUDT6	NUDT4, NUDT5, NUDT7, NUDT8, NUDT9
NUDT7	NUDT4, NUDT5, NUDT6, NUDT8, NUDT9
NUDT8	NUDT4, NUDT5, NUDT6, NUDT7, NUDT9
NUDT9	NUDT4, NUDT5, NUDT6, NUDT7, NUDT8
NUDT10	NUDT11, NUDT17, NUDT19, NUDT21
NUDT11	NUDT10, NUDT17, NUDT19, NUDT21
NUDT12	NUDT13
NUDT13	NUDT12
NUDT14	NUDT15, NUDT16
NUDT15	NUDT14, NUDT16
NUDT16	NUDT14, NUDT15
NUDT17	NUDT10, NUDT11, NUDT19, NUDT21
NUDT18	DCP2
NUDT19	NUDT10, NUDT11, NUDT17, NUDT21
DCP2	NUDT18
NUDT21	NUDT10, NUDT11, NUDT17, NUDT19
NUDT22	

h

	**Alleviating gene Interactions (95% confidence interval)**				**Aggravating gene Interactions (95% confidence interval)**			
	CCD841	SW480	MCF7	A549	CCD841	SW480	MCF7	A549
NUDT1			NUDT11				NUDT6	
NUDT2								
NUDT3		NUDT11	NUDT11, NUDT21	NUDT11, NUDT22	DCP2			
NUDT4		NUDT11						
NUDT5		NUDT11					DCP2	
NUDT6					NUDT18, DCP2		NUDT1, NUDT12, NUDT17, NUDT18, DCP2	NUDT8, NUDT12, NUDT18
NUDT7		NUDT11						
NUDT8						NUDT15		NUDT6, NUDT12
NUDT9		NUDT11	NUDT14					
NUDT10	NUDT21	NUDT21						
NUDT11		NUDT3, NUDT4, NUDT5, NUDT7, NUDT9, NUDT14, NUDT17, NUDT19, NUDT21	NUDT1, NUDT3, NUDT14	NUDT3				
NUDT12					DCP2		NUDT6, NUDT17	NUDT6, NUDT8, NUDT18, DCP2
NUDT13								
NUDT14		NUDT11	NUDT9, NUDT11, NUDT16, NUDT19	NUDT19, NUDT22				
NUDT15						NUDT8		
NUDT16			NUDT14					
NUDT17							NUDT6, NUDT17, DCP2	
NUDT18							NUDT6	NUDT6, NUDT12
NUDT19		NUDT11	NUDT14		DCP2			
DCP2					NUDT3, NUDT6, NUDT12, NUDT19		NUDT5, NUDT6, NUDT17	NUDT12
NUDT21	NUDT10	NUDT22	NUDT3	NUDT19				
NUDT22		NUDT21		NUDT3, NUDT14, NUDT19				

i

	**mRNA expression in cancer (p-value<0.001)**	
	Up-regulated	Down-regulated
NUDT1	DLBC, COAD, SARC, MESO, LUSC, CHOL, TGCT, BLCA	-
NUDT2	DLBC, MESO, TGCT	-
NUDT3	COAD, UCEC, UCS, CHOL	SARC
NUDT4	-	ACC, PCPG, DLBC, COAD, UCEC, UCS, CESC, SARC, CHOL, MESO, LUAD, LUSC, OV, PAAD, READ, TGCT
NUDT5	ACC, DLBC, UCEC, UCS, CESC, SARC, CHOL, MESO, OV, PAAD, TGCT, BLCA	-
NUDT6	-	COAD, MESO, KIRC, TGCT
NUDT7	-	UCEC, UCS, MESO, TGCT
NUDT8	DLBC, MESO	SARC
NUDT9	ACC, CHOL, SKCM	CESC, CHOL
NUDT10	SARC, LUSC, MESO (Lung)	ACC, UCEC, CESC, CHOL, TGCT, MESO (Testis)
NUDT11	LUSC, MESO (Lung), HNSC	ACC, UCEC, CESC, SARC, KIRP, TGCT, MESO (Testis)
NUDT12	-	DLBC, COAD, UCEC, UCS, CESC, MESO, KIRC, KIRP, LUAC, LUSC, READ, TGCT
NUDT13	CHOL	DLBC, UCS, CESC, LUSC, PAAD, SKCM, TGCT, MESO
NUDT14	DLBC, TGCT	-
NUDT15	LUSC, TGCT	ACC, PCPG
NUDT16	-	-
NUDT17	BLCA	-
NUDT18	MESO	TGCT
NUDT19	SARC, OV, MESO	PAAD, CHOL
DCP2	UCS, SAC, MESO, TGCT	-
NUDT21	-	ACC, PCPG, MESO
NUDT22	DLBC, KIRP, HNSC, TGCT, MESO	-

Supplementary Figure 17

Supplementary Figure 17: Results summary tables for each of the datasets obtained in our study. A: Structure similarity. B: Substrate activity. C: Protein expression in normal tissues. D: Survival upon siRNA-meditated knockdown. E: Sub-cellular localisation. F: Genetic interactions inferred by Réd. G: Clusters determined by FUSION. H: Alleviating or Aggravating epistatic interactions and I: mRNA expression in cancer (TCGA).

Supplementary Tables

**Normal tissue**	**Tumor**
Adrenal gland	Adrenocortical carcinoma
	Pheochromocytoma and Paraganglioma
Bone marrow	Acute Myeloid Leukemia
	Lymphoid Neoplasm Diffuse Large B-cell Lymphoma
Brain (cerebral cortex)	Brain Lower Grade Glioma
	Glioblastoma multiforme
Colon	Colon adenocarcinoma
Duodenum	Colon adenocarcinoma
Endometrium	Uterine Corpus Endometrial Carcinoma
	Uterine Carcinosarcoma
	Cervical squamous cell carcinoma and endocervical adenocarcinoma
Adipose tissue	Sarcoma
Gall bladder	Cholangiocarcinoma
Heart muscle	Mesothelioma
Kidney	Kidney renal clear cell carcinoma
	Kidney renal papillary cell carcinoma
Liver	Liver hepatocellular carcinoma
Lung	Lung adenocarcinoma
	Lung squamous cell carcinoma
	Mesothelioma
Lymphnode	Lymphoid Neoplasm Diffuse Large B-cell Lymphoma
Ovary	Ovarian serous cystadenocarcinoma
Pancreas	Pancreatic adenocarcinoma
	Cholangiocarcinoma
Prostate	Prostate adenocarcinoma
Rectum	Rectal adenocarcinoma
Salivary gland	Head and Neck squamous cell carcinoma
Skeletal muscle	Sarcoma
Skin	Skin Cutaneous Melanoma
Smooth muscle	Sarcoma
Testis	Testicular Germ Cell Tumors
	Mesothelioma
Thyroid	Thyroid carcinoma
Urinary bladder	Bladder Urothelial carcinoma

Supplementary Table 1: Pairwise comparisons between normal tissues and tumors. This pairing was used to match normal and cancer tissues for the analysis of TCGA and HPA sequencing data.

Data set	Data set position in the fusion graph	Data set description	Data set size
R(1,2)	R(NUDIX genes, genes in the HPA)	Spearman rank correlation measurements of mRNA coexpression in the HPA data	22x978
R(1,3)	R(NUDIX genes, TCGA cancer projects)	mRNA expression in cancer tissues taken from the TCGA database	22x29
R(1,4)	R(NUDIX genes, genes in the COAD cohort)	Spearman rank correlation measurements of mRNA coexpression in the COAD cohort	22x1891
R(1,5)	R(NUDIX genes, genes in the LUSC cohort)	Spearman rank correlation measurements of mRNA coexpression in the LUSC cohort	22x141
R(1,6)	R(NUDIX genes, genes in the LUAD cohort)	Spearman rank correlation measurements of mRNA coexpression in the LUAD cohort	22x335
R(1,7)	R(NUDIX genes, genes in the BRCA cohort)	Spearman rank correlation measurements of mRNA coexpression in the BRCA cohort	22x909
R(1,8)	R(NUDIX genes, measurements of area in SW480 cells)	Cell area of single siRNA knockdowns in the SW480 cells obtained with Cell Profiler software	22x100
R(1,9)	R(NUDIX genes, measurements of area in A549 cells)	Cell area of single siRNA knockdowns in the A549 cells obtained with Cell Profiler software	22x100
R(1,10)	R(NUDIX genes, measurements of area in MCF7 cells)	Cell area of single siRNA knockdowns in the MCF7 cells obtained with Cell Profiler software	22x100
R(1,11)	R(NUDIX genes, measurements of eccentricity in A549 cells)	Cell eccentricity of single siRNA knockdowns in the A549 cells obtained with Cell Profiler software	22x100
R(1,12)	R(NUDIX genes, measurements of eccentricity in MCF7 cells)	Cell eccentricity of single siRNA knockdowns in the MCF7 cells obtained with Cell Profiler software	22x100
R(1,13)	R(NUDIX genes, measurements of eccentricity in SW480 cells)	Cell eccentricity of single siRNA knockdowns in the SW480 cells obtained with Cell Profiler software	22x100
R(1,14)	R(NUDIX genes, measurements of cell cycle in MCF7 cells)	Counts of single siRNA knockdown cells in the MCF7 across cell cycle phases <2N, 2N, S, 4N, >4N obtained with Cell Profiler software	22x5
R(1,15)	R(NUDIX genes,	Counts of single siRNA knockdown	22x5

	measurements of cell cycle in A549 cells)	cells in the A549 across cell cycle phases <2N, 2N, S, 4N, >4N obtained with Cell Profiler software	
R(1,16)	R(NUDIX genes, measurements of cell cycle in SW480 cells)	Counts of single siRNA knockdown cells in the SW480 across cell cycle phases <2N, 2N, S, 4N, >4N obtained with Cell Profiler software	22x5
R(1,1)	R(NUDIX genes, NUDIX genes)	Cell area of double siRNA knockdowns in the A549 cells obtained with Cell Profiler software	22x22x100
R(1,1)	R(NUDIX genes, NUDIX genes)	Cell area of double siRNA knockdowns in the SW480 cells obtained with Cell Profiler software	22x22x100
R(1,1)	R(NUDIX genes, NUDIX genes)	Cell area of double siRNA knockdowns in the MCF7 cells obtained with Cell Profiler software	22x22x100
R(1,1)	R(NUDIX genes, NUDIX genes)	Cell eccentricity of double siRNA knockdowns in the A549 cells obtained with Cell Profiler software	22x22x100
R(1,1)	R(NUDIX genes, NUDIX genes)	Cell eccentricity of double siRNA knockdowns in the SW480 cells obtained with Cell Profiler software	22x22x100
R(1,1)	R(NUDIX genes, NUDIX genes)	Cell eccentricity of double siRNA knockdowns in the MCF7 cells obtained with Cell Profiler software	22x22x100
R(1,1)	R(NUDIX genes, NUDIX genes)	Counts of double siRNA knockdown cells in the A549 across cell cycle phases <2N, 2N, S, 4N, >4N obtained with Cell Profiler software	22x22x5
R(1,1)	R(NUDIX genes, NUDIX genes)	Counts of double siRNA knockdown cells in the SW480 across cell cycle phases <2N, 2N, S, 4N, >4N obtained with Cell Profiler software	22x22x5
R(1,1)	R(NUDIX genes, NUDIX genes)	Counts of double siRNA knockdown cells in the MCF7 across cell cycle phases <2N, 2N, S, 4N, >4N obtained with Cell Profiler software	22x22x5
R(1,1)	R(NUDIX genes, NUDIX genes)	Epistasis score of interaction in the A549 cells	22x22
R(1,1)	R(NUDIX genes, NUDIX	Epistasis score of interaction in the	22x22

	genes)	SW480 cells	
R(1,1)	R(NUDIX genes, NUDIX genes)	Epistasis score of interaction in the MCF7 cells	22x22

Color legend	
	Data from the MCF7 human breast cancer cells
	Data from the A549 human lung cancer/adenocarcinoma cells
	Data from the SW480 human colon adenocarcinoma cells
	Data not specific to any individual cancer tissue or cell line

Supplementary Table 2: List of data sets used for clustering the human NUDIX family.

**Cancer name**	**Abbreviation.**
Adrenocortical carcinoma	ACC
Bladder Urothelial Carcinoma	BLCA
Breast invasive carcinoma	BRCA
Cervical squamous cell carcinoma and endocervical adenocarcinoma	CESC
Cholangiocarcinoma	CHOL
Colon adenocarcinoma	COAD
Lymphoid Neoplasm Diffuse Large B-cell Lymphoma	DLBC
Glioblastoma multiforme	GBM
Head and Neck squamous cell carcinoma	HNSC
Kidney renal clear cell carcinoma	KIRC
Kidney renal papillary cell carcinoma	KIRP
Acute Myeloid Leukemia	LAML
Brain Lower Grade Glioma	LGG
Liver hepatocellular carcinoma	LIHC
Lung adenocarcinoma	LUAD
Lung squamous cell carcinoma	LUSC
Mesothelioma	MESO
Ovarian serous cystadenocarcinoma	OV
Pancreatic adenocarcinoma	PAAD
Pheochromocytoma and Paraganglioma	PCPG
Prostate adenocarcinoma	PRAD
Rectum adenocarcinoma	READ
Sarcoma	SARC
Skin Cutaneous Melanoma	SKCM
Testicular Germ Cell Tumors	TGCT
Thyroid carcinoma	THCA
Uterine Corpus Endometrial Carcinoma	UCEC
Uterine Carcinosarcoma	UCS
Uveal Melanoma	UVM

Supplementary Table 3: TCGA cancer types abbreviations.

Gene	Distributor	Product code	Species	Dilution	Ensembl Gene ID
MTH1/ NUDT1	Atlas Antibodies AB	HPA012636	Rabbit	300	ENSG00000106268
NUDT5	Atlas Antibodies AB	HPA019827	Rabbit	250	ENSG00000165609
NUDT7	Atlas Antibodies AB	HPA042042	Rabbit	35	ENSG00000140876
NUDT8	Atlas Antibodies AB	HPA041466	Rabbit	250	ENSG00000167799
NUDT9	Atlas Antibodies AB	HPA044866	Rabbit	3000	ENSG00000170502
NUDT12	Atlas Antibodies AB	HPA045449	Rabbit	800	ENSG00000112874
NUDT13	Atlas Antibodies AB	HPA040636	Rabbit	35	ENSG00000166321
NUDT14	Atlas Antibodies AB	HPA046755	Rabbit	150	ENSG00000183828
NUDT15	Atlas Antibodies AB	HPA038969	Rabbit	1250	ENSG00000136159
NUDT16	Atlas Antibodies AB	HPA060452	Rabbit	15	ENSG00000198585
NUDT17	Atlas Antibodies AB	HPA030145	Rabbit	500	ENSG00000186364
NUDT18	Atlas Antibodies AB	HPA028581	Rabbit	10	ENSG00000275074
NUDT22	Atlas Antibodies AB	HPA039334	Rabbit	40	ENSG00000149761
DCP2	Abcam plc	ab28658	Rabbit	35	ENSG00000172795

Supplementary Table 4: List of antibodies used for TMA and IHC.

Gene Symbol	GENE ID	Gene Accession	siRNA Sequence
NUDT1	4521	NM_002452	GACGACAGCUACUGGUUUC
NUDT1	4521	NM_002452	GAAAUUCCACGGGUACUUC
NUDT1	4521	NM_002452	CGACGACAGCUACUGGUUU
NUDT1	4521	NM_002452	AGACGUGGCUGCUGAACAG
NUDT2	318	NM_001161	GAGGGAGACCCAAGAGGAA
NUDT2	318	NM_001161	GAGCAUGUGGCUUGAUCAU
NUDT2	318	NM_001161	UUCCCAAAGUGGACAACAA
NUDT2	318	NM_001161	GAAGAUGCCUCAUUCCCAA
NUDT3	11165	NM_006703	GCACAGGACGUAUGUCUAU
NUDT3	11165	NM_006703	GAAGGAAGAGGGAAUGGUU
NUDT3	11165	NM_006703	UAAAAGUGCUGCAGUAUCA
NUDT3	11165	NM_006703	UGAGGAGGCUGGAGUAAAA
NUDT4	11163	NM_019094	GGAUAAUAAUGCCUUGUUU
NUDT4	11163	NM_019094	GAAUGGAACCCGAGGAGGA
NUDT4	11163	NM_019094	CGAAAGCACAGAACAUAUG
NUDT4	11163	NM_019094	GGGUUGCCAUCUAGUGUAA
NUDT5	11164	NM_014142	GCAGAGAACACUUCACUAU
NUDT5	11164	NM_014142	GGCAAACAGUAUAUCAUUU
NUDT5	11164	NM_014142	CAGAGGAGUUAAUUUCAGA
NUDT5	11164	NM_014142	GAACUUGGGAAUCAGUGAA
NUDT6	11162	NM_007083	GAAGAAUGCUUAAGAUGUG
NUDT6	11162	NM_007083	GAACUGCCAGAGAAUUAUA
NUDT6	11162	NM_007083	UAUAUCAUCUGCCGCCUAA
NUDT6	11162	NM_007083	CCAUAUUCAUUCACCAUAA
NUDT7	283927	NM_001105663	GUAUGAUAUUGGAGGCAAA
NUDT7	283927	NM_001105663	UCAAGGGAAUGACGGCAAA
NUDT7	283927	NM_001105663	ACAAAGAACUAUUCACGAG
NUDT7	283927	NM_001105663	GCCAUGUCUUAUUGAUACA
NUDT8	254552	NM_181843	GCACAAGGGCGACGUCAGU
NUDT8	254552	NM_181843	GGUGUAGGCCCACUGGAUC
NUDT8	254552	NM_181843	ACUCGGAGGAGGUGAGCUG
NUDT8	254552	NM_181843	CUGUGUAUGAUCCGCAAAA
NUDT9	53343	NM_024047	GGAAGCAUAUCUUACAAUU
NUDT9	53343	NM_024047	GAAGCCAGGUUCCUAAUGA
NUDT9	53343	NM_024047	GAACUACCAUGACGAAACA
NUDT9	53343	NM_024047	GGAUAGCAGUGGAAAUAAA
NUDT10	170685	NM_153183	GCACAGAACGUACGUGUAU
NUDT10	170685	NM_153183	GACGAGGUCCUGUUAGUGA
NUDT10	170685	NM_153183	CGCCGAAUAUCUGGAGAAA
NUDT10	170685	NM_153183	GUGCAAACCCAACCAGACA
NUDT11	55190	NM_018159	CCGAAUAUCUGGAGAAACU
NUDT11	55190	NM_018159	GCACAGAACGUACGUGUAU
NUDT11	55190	NM_018159	GGGAAGAUUCGGUUAGCAU
NUDT11	55190	NM_018159	GGAGCGAACGCGAGGACGA
NUDT12	83594	NM_031438	CAUAAUACCUCAUACCCAA
NUDT12	83594	NM_031438	GGUUGUAGCUCAAGCAAGA
NUDT12	83594	NM_031438	GGAGAUAUUGCCAAGUUAA
NUDT12	83594	NM_031438	GGAAAGUGGAGUCAAAGUU
NUDT13	25961	NM_015901	GCACAAAGAAUAGAAGAUU

NUDT13	25961	NM_015901	UGGAAAGGCUCCUGGGUAA
NUDT13	25961	NM_015901	GGAAUAGCUUGCAGGAGAA
NUDT13	25961	NM_015901	CAACCUAUGUUACUAAGAC
NUDT14	256281	NM_177533	GGACGGGCCUCGGGAGCUA
NUDT14	256281	NM_177533	UGGAGGAAGUGGCUUGCAA
NUDT14	256281	NM_177533	GGAGCCUGGUGUUGGUGAA
NUDT14	256281	NM_177533	AGACAGACCAUGUUCUACA
NUDT15	55270	NM_018283	GGAUGUGACUCAUGAUUCA
NUDT15	55270	NM_018283	ACAAGGCUAUGAUCCAUUU
NUDT15	55270	NM_018283	GGAGAAUUACCAUUAUGUU
NUDT15	55270	NM_018283	CAUCUGGAGUUCGGUGAAA
NUDT16	131870	NM_152395	GCUGCAGUCUGGCUCUAUU
NUDT16	131870	NM_152395	CUACCUUCCUGGAGAAUUC
NUDT16	131870	NM_152395	CUGUAUACCCUGCGGGAUG
NUDT16	131870	NM_152395	CCUUAAGAUUCCAGCUCAU
NUDT17	200035	NM_001012758	UCUGGAAAGUGAAGUGUAA
NUDT17	200035	NM_001012758	GGACGGAGGGCUUCGAGAA
NUDT17	200035	NM_001012758	CAACCUUUAUUAUGGGUGA
NUDT17	200035	NM_001012758	GUUUAUGGGAGUCUGCCUA
NUDT18	79873	NM_024815	GUGGAAUUCUCAAGACUUC
NUDT18	79873	NM_024815	GAGCUACCCUGUGAUCUGG
NUDT18	79873	NM_024815	GCAAGAGGCUGGCGAUCAG
NUDT18	79873	NM_024815	GCACGUUGCUGAGUAAAGA
NUDT19	390916	NM_001105570	UAAGAACUCUGUAGUAAGA
NUDT19	390916	NM_001105570	UGAGAAGACUUGCAAACUU
NUDT19	390916	NM_001105570	GACAUACCAUCGCCACCUU
NUDT19	390916	NM_001105570	CCACGUGACUGUUCAGCCA
DCP2	167227	NM_152624	GAAAUUGCCUUGUCAUAGA
DCP2	167227	NM_152624	GAUAAGAGACUUUGCUAAA
DCP2	167227	NM_152624	GAAGAGAAAUUCGGAACAU
DCP2	167227	NM_152624	GGAAACUUCAGGAUAAUUU
NUDT21	11051	NM_007006	GCGCAUGAGGGAAGAAUUU
NUDT21	11051	NM_007006	CUAAGGAACAUAAGAAGUU
NUDT21	11051	NM_007006	UGACAAUGCACCAGGAUAU
NUDT21	11051	NM_007006	ACGCUUAAUGACAGAGAUA
NUDT22	84304	NM_032344	CCACGAGUCUACAGGAAUC
NUDT22	84304	NM_032344	GCCGAUGACUUCCUUGUCU
NUDT22	84304	NM_032344	ACACAGAACGUGCGGAGAU
NUDT22	84304	NM_032344	GAGCCAGUGCCGAGUUCUA

Gene Symbol house keeping genes	qRT-PCR primer sequence 5'-3'
hGAPDH forward	AAGGTCGGAGTCAACGGATT
hGAPDH reverse	CTCCTGGAAGATGGTGATGG
Beta Actin forward	CCTGGCACCCAGCACAAT
Beta Actin reverse	GGCCGGACTCGTCATACT

Gene Symbol	GENE ID	Gene Accession	qRT-PCR primer sequence 5'-3'
NUDT1 forward	4521	NM_002452.3	GGTGCAGAACCCAGGGAC
NUDT1 reverse	4521	NM_002452.3	CTTTGCCCCCAAAGCCATTC
NUDT2 forward	318	NM_001161.4	TTTCTGCCTCAACCACGGAC

NUDT2 reverse	318	NM_001161.4	CAAACGCACCCATTCCAGAAG
NUDT3 forward	11165	NM_006703.3	AGCTCAAGTCGAACCAGACC
NUDT3 reverse	11165	NM_006703.3	TCACGAGTAGCACCTCCTCC
NUDT4 forward	4521	NM_002452	CCAGTGGATTGTCCCAGGAG
NUDT4 reverse	4521	NM_002452	GGTCTTGGTTCTCAAATATGCCC
NUDT5 forward	11164	NM_014142	ATCCTTTTAGCACCGCGAGA
NUDT5 reverse	11164	NM_014142	CAGGTGAGAAGTTCACCTCCAA
NUDT6 forward	11162	NM_007083	GTAGGAGTTGCAGGAGCTGT
NUDT6 reverse	11162	NM_007083	CTTCTCGAACCGCTGTGTCT
NUDT7 forward	283927	NM_001105663	CCGTCCTTTTGCCATTGGTG
NUDT7 reverse	283927	NM_001105663	GGGGCCCTTCTTAGCTTCTC
NUDT8 forward	254552	NM_181843	CCCAACTCGGAGGAGGTAGA
NUDT8 reverse	254552	NM_181843	CCATGCAGGAAGACGGGTAG
NUDT9 forward	53343	NM_024047	ACCTAGCATCGCAAAGCCG
NUDT9 reverse	53343	NM_024047	ACGAGTTTCTGAACGCCTGG
NUDT10 forward	170685	NM_153183.2	CGTGCCACACACCTCGG
NUDT10 reverse	170685	NM_153183.2	TGTCTGGTTGGGTTTGCACT
NUDT11 forward	55190	NM_018159.3	AGCGATCCCTAATGAACAGCA
NUDT11 reverse	55190	NM_018159.3	TGGGAACACAGAAGTGCTCA
NUDT12 forward	83594	NM_031438.2	CTGTGGTCACCTTTGGGAAGA
NUDT12 reverse	83594	NM_031438.2	GGCAATATCTCCTTCAGCAGC
NUDT13 forward	25961	NM_015901.5	AGCCGTTGAACGTGAACATTT
NUDT13 reverse	25961	NM_015901.5	TCTCCTGCAAGCTATTCCACA
NUDT14 forward	256281	NM_177533.4	CATTACCGCCAGAATGGTGC
NUDT14 reverse	256281	NM_177533.4	GGCTCCTCCGAGAAGAGTTG
NUDT15 forward	55270	NM_018283.1	GGAAGAGGAAAGGCTCGGTT
NUDT15 reverse	55270	NM_018283.1	CCCAGGTTTCACCGAACTCC
NUDT16 forward	131870	NM_001171906.1	GTGCCACGCTCTCCTCTAC
NUDT16 reverse	131870	NM_001171906.1	GCATCTGCATCAGTATGGCG
NUDT17 forward	200035	NM_001012758.2	GAGTGTGTGTGGCCTCCTG
NUDT17 reverse	200035	NM_001012758.2	AGAAAGGGGGTCGCTGGA
NUDT18 forward	79873	NM_024815.3	GGCTGCGGAAGAACGTGT
NUDT18 reverse	79873	NM_024815.3	GATCAGTAGCACCTCATCCTGC
NUDT19 forward	390916	NM_001105570.1	CTCACCCCTTTCTTGCGGG
NUDT19 reverse	390916	NM_001105570.1	CTGATGGAGATGACCACTGGTAG
NUDT20 forward	167227	NM_152624.5	CTGACGGTTCTCCTGGTGAC
NUDT20 reverse	167227	NM_152624.5	TCCCCTCATACTTTGATTCTTTCCT
NUDT21 forward	11051	NM_007006.2	GCACCATCAACCTGTACCCT
NUDT21 reverse	11051	NM_007006.2	CTCATGCGCTGAAATCTGGC

Supplementary Table 5: List of siRNA sequences used for the depletion of all the NUDIX genes, and list of qRT-PCR primers used to determine NUDIX mRNA levels.

Supplementary Note:

Methodology remarks

The methodologies used in this work are a combination of well-established approaches as well as innovative bioinformatics analyses. Despite our careful assessment, execution and interpretation of experiments and results, there are limitations to take into consideration.

The outcome of the structural analysis based on phylogeny may vary depending on the method applied, indeed, the combination of Markov chain Monte Carlo (MCMC) and Bayesian algorithms, gave the most statistically robust phylogenetic tree, which differed from other methods, thus a careful choice of method is of utmost importance.

We used the Malachite Green assay to determine substrate activity for the 18 NUDIX enzymes that we analysed. Given the large diversity of substrates and enzymes, the conditions in which the highest hydrolase activity is achieved are equally diverse. We opted for a unified condition in the presence of magnesium and a constant pH of 7.5, which may have limited the efficiency of the reactions, therefore limiting the number of identified substrates. The limitations of single point substrate activity assays are to be considered, as well as the relevancy of such assessments regarding the availability of the chosen substrates in the cell, as recently reported[1], since the concentration of the identified substrates may, in some cases, be considerably lower in cells than the concentration used in our substrate screen. Also, the concentration

of the individual NUDIX proteins in the cell is not known, hence, our results should serve as a basis for more in depth functional studies of this family of proteins. The complete lack of activity observed for some NUDIX hydrolases may indicate the need of specific reaction conditions and/or absence of their substrates in the tested panel. For example, NUDT10 and NUDT11 may require manganese as divalent cation instead of magnesium as has been previously suggested[2]. Another possibility is that the His-tag, present on all of our purified NUDIX proteins, in some cases may negatively affect the enzyme activity. Finally, murine NUDT7 has been shown to hydrolyse Coenzyme A[3], an activity we could not reproduce. The lack of activity of NUDT6 and NUDT21 is consistent with earlier publications[4, 5]

The TCGA as well as the Human Protein Atlas databases contain a plethora of valuable data, however to make use of it, especially when comparing datasets from two different databases, strict normalizations need to be made. Our normalized data clearly indicates differences among the different NUDIX at mRNA and protein levels, in both cancer and normal tissues, however, the experimental conditions in which these measurements have been made may condition the results, thus individual validation may be advisable for further studies.

Despite the risk of potential off-target effects, small interference RNAs are widely used for the transient depletion of target genes. We used this approach to evaluate the viability of the studied cell lines in the absence of the NUDIX enzymes, as well as to investigate potential epistatic relationships among these. We evaluated the overall knockdown efficiency by qPCR and statistically compared two independent single siRNA-mediated depletion experiments, which highly correlated, validating our approach. However, it is important to note that the potential epistatic interactions could be masked by inefficient knockdown in a given population of cells. For epistasis analysis, we considered single and double knockdown viability measurements, and for a pair of NUDIX enzymes, we analysed whether a change in the first gene

masked the effects of a knockdown in the second gene. We used the multiplicative model to calculate the expected viability of a double knockdown in the absence of a genetic interaction. Although the multiplicative model is an established approach to define genetic interaction and was previously used in many large-scale genetic interactions studies, other mathematically distinct definitions of genetic interactions are possible, including the additive and logarithmic definitions. We thus report quantitative viability measurements of single and double NUDIX knockdowns to allow reclassification of interactions under any definition of genetic interaction.

To infer the order of function of the NUDIX enzymes in molecular pathways that are active in different cell lines we used a probabilistic approach to epistasis-based gene network inference. This approach, called Réd, allowed us to assemble a NUDIX gene network in each cell line based on probabilistic scoring of gene relationships measured in that cell line. Previous *in-silico* analyses showed that Réd can accurately reconstruct known gene pathways, is robust to noise and measurement errors, and that it outperforms current methods for gene network reconstruction. However, inference of gene networks requires solving a complicated non-convex optimization problem and current optimization techniques can only find a local mathematical optimum of the optimization problem through application of gradient descent algorithms. We thus performed additional computational experiments and sensitivity analyses to test the inferred gene networks and to validate their robustness to small data perturbations and to variation of optimization parameters.

Our integrative analyses used the FUSION algorithm to cluster NUDIX enzymes based on joint consideration of 27 datasets. The considered datasets are heterogeneous, differ in scale and range of values, and contain diverse measurements to describe qualitatively different phenomena relevant to the NUDIX enzymes. Therefore our analysis involved careful normalization of each dataset and its representation with a data matrix, followed by an

application of the machine learning algorithm FUSION, that inferred a predictive latent model, and clustering of the NUDIX enzymes based on the inferred model. To test the quality of the inferred model we tracked the progress of optimization implemented in the FUSION algorithm. We validated the results by experimentally interrogating selected NUDIX clusters identified by the FUSION algorithm, performing siRNA-mediated depletion of the NUDIX genes assigned to a given cluster, and evaluated the effect on expression of the rest of the NUDIX enzymes present in the cluster by qPCR.

Obtaining the sub-cellular localization of each NUDIX was not an objective of this work, for this we referred to the publicly available databases Human Protein Atlas and UniProt. It is important to note that the information in these databases is updated as measurements become more accurate and information becomes available; therefore certain localisations here mentioned might be subjected to changes. Despite the potential specific drawbacks and limitations of each methodology, we have applied normalization approaches to minimize errors and variations, providing an overall high confidence in the herein presented results.

References

1. Nguyen, V.N. *et al.* Substrate specificity characterization for eight putative nudix hydrolases. Evaluation of criteria for substrate identification within the Nudix family. *Proteins* **84**, 1810-1822 (2016).
2. Leslie, N.R., McLennan, A.G. & Safrany, S.T. Cloning and characterisation of hAps1 and hAps2, human diadenosine polyphosphate-metabolising Nudix hydrolases. *BMC Biochem.* **3**, 20 (2002).
3. Gasmi, L. & McLennan, A.G. The mouse Nudt7 gene encodes a peroxisomal nudix hydrolase specific for coenzyme A and its derivatives. *Biochem J* **357**, 33-38 (2001).
4. Baguma-Nibasheka, M., Li, A.W. & Murphy, P.R. The fibroblast growth factor-2 antisense gene inhibits nuclear accumulation of FGF-2 and delays

cell cycle progression in C6 glioma cells. *Mol Cell Endocrinol* **267**, 127-136 (2007).

5. Yang, Q., Gilmartin, G.M. & Doublie, S. Structural basis of UGUA recognition by the Nudix protein CFI(m)25 and implications for a regulatory role in mRNA 3' processing. *Proc Natl Acad Sci U S A* **107**, 10062-10067 (2010).
