# Supplementary material for: A comprehensive structural, biochemical and biological profiling of the human NUDIX hydrolase family
Source: Nat Commun. 2017 Nov 16;8:1541. doi: 10.1038/s41467-017-01642-w (PMC5688067; doi:10.1038/s41467-017-01642-w)
Supplement: Supplementary file 3 — Description of Additional Supplementary Files [file 41467_2017_1642_MOESM3_ESM.pdf]

Descriptions of Additional Supplementary File

File Name: Supplementary Data 1
Description: NUDIX pairs for each epistasis score bin

File Name: Supplementary Data 2
Description: Cell cycle distributions measured by PopulationProfiler upon double and single siRNA-mediated depletion, in CCD841, A549, MCF7 and SW480 cells
